# An Augmented Reality-Based Navigation System for Stereotactic Brain Biopsy with Multi-Objective Path Planning and Hybrid Registration

**DOI:** 10.3390/brainsci16030296

**Published:** 2026-03-06

**Authors:** Tao Zhang, Shuyi Wang, Yueyang Zhong, Haoliang Li, Jingyi Hu, Haokun Wang

**Affiliations:** School of Health Science and Engineering, University of Shanghai for Science and Technology, Shanghai 200093, China; 233352437@st.usst.edu.cn (T.Z.); 233352436@st.usst.edu.cn (Y.Z.); 232322256@st.usst.edu.cn (H.L.); 232322248@st.usst.edu.cn (J.H.); 243352425@st.usst.edu.cn (H.W.)

**Keywords:** augmented reality, automated path planning, stereotactic brain biopsy, neuronavigation

## Abstract

**Highlights:**

**What are the main findings?**

- A safety-oriented multi-objective optimization model enables automated and clinically compliant biopsy path planning.
- A hybrid registration strategy combining QR codes and anatomical landmarks achieves accurate augmented reality alignment with rapid clinical setup.

**What are the implications of the main findings?**

- Explicitly embedding clinical constraints into automated path planning reduces reliance on manual decision-making during preoperative surgical planning.
- Intuitive, real-time three-dimensional overlays of anatomical structures and planned paths can reduce surgeons’ cognitive load in intraoperative neuronavigation.

**Abstract:**

Background: Stereotactic brain biopsy is the gold standard for the pathological diagnosis of malignant brain tumors. However, conventional procedures rely heavily on manual path planning and unintuitive navigation, which significantly increase the risk of severe complications and impose an additional cognitive burden on surgeons. Methods: We propose an augmented reality-based navigation system that synergizes multi-objective path planning with hybrid registration. Preoperatively, the system utilizes a constrained multi-objective optimization (MOO) model derived from clinical criteria to automatically calculate and visualize optimal biopsy paths within a three-dimensional anatomical environment. Intraoperatively, the system performs rapid initial alignment using quick response (QR) codes, followed by precise refinement through anatomical landmarks. This process ultimately enables the highly accurate, real-time overlay of the surgical path and anatomical models onto the patient’s operative field. Results: An expert study across four common brain tumor locations demonstrated that the MOO model significantly outperformed manual methods in satisfying safety criteria. The hybrid registration reduced the mean fiducial registration error (FRE) from 4.19 ± 1.11 mm to 2.37 ± 0.91 mm (*p* < 0.001), with a mean target registration error (TRE) of 2.34 ± 0.71 mm and a mean clinical setup time of 2.63 ± 0.36 min. Conclusions: This system assists stereotactic brain biopsy through automated path planning and immersive augmented reality-based guidance, highlighting its potential to support surgical workflow consistency and procedural safety.

## 1. Introduction

The worldwide incidence rate of primary brain tumors is 10.82 instances per 100,000 person-years, with malignant brain tumors constituting roughly 25.32% of these cases [1]. Precise pathological diagnosis is essential for treatment planning, and stereotactic brain biopsy is recognized as the gold standard for acquiring pathological tissue. This procedure typically utilizes magnetic resonance imaging (MRI) guidance to ensure safe and accurate navigation to the target lesion. Notwithstanding its significant diagnostic value, stereotactic brain biopsy entails inherent risks. The most common complications associated with this procedure include the necessity for a further biopsy and intracranial hemorrhage, which can lead to severe consequences [2]. Therefore, the key objective of surgical planning is to enhance the accuracy of lesion location while avoiding critical structures to reduce the incidence of intraoperative complications.

Frame-based stereotactic navigation is the standard procedure for deep lesion biopsy [3]. However, the lengthy frame installation process complicates the procedure and increases patient pain. Once the instrument is set, any movement of the patient’s head within the frame reduces navigation accuracy. Frameless navigation using optical or electromagnetic tracking systems offers an alternative approach to stereotactic biopsy. Nevertheless, it requires surgeons to redirect their visual attention from the surgical area to a display, consequently increasing the surgeon’s cognitive load [4].

Augmented reality (AR) can overlay three-dimensional (3D) anatomical models and surgical plans onto the operative field through head-mounted displays (HMDs), facilitating a more intuitive approach for physicians during procedures. Several studies have investigated AR-based navigation systems for brain biopsy. Satoh et al. [5] developed the Trans-visible navigator for tablet applications. The system utilizes a motion capture unit to register anatomical models to the patient by anatomical landmarks, assisting surgeons in determining drill positions and puncture paths. However, the study merely demonstrated the clinical feasibility of AR-based navigation in frame-based stereotactic brain biopsy, without providing rigorous experimental validation. Majak et al. [6] and Gibby et al. [7] proposed AR-based navigation systems with HMDs. Surgeons are able to concurrently observe the surgical field and preplanned paths through the HMD. Their approaches achieved accuracy errors of 3.62 ± 1.71 mm and 2.50 ± 0.93 mm in phantom experiments, sufficient for ventriculostomy or large lesion biopsy but not for small targets [8]. Nevertheless, current AR-based navigation systems primarily rely on marker-based 3D registration methods (e.g., reflective spheres, quick response codes), which require additional markers to be fixed to the patient preoperatively. Since the relative position between markers and the 3D model is predefined, improper marker placement reduces navigation accuracy. Zhou et al. [9] introduced a markerless registration method. The optical sensors of HoloLens 2 continually acquire the 3D coordinates of the patient’s skin surface by tracking a handheld probe. A point cloud registration algorithm is then applied to align the 3D anatomical model with the patient, achieving a mean registration error of 1.86 mm. However, the process requires the surgeon to execute multiple interactive acquisition steps, resulting in a mean registration time of 7.95 min. Consequently, the development of an efficient and high-accuracy 3D registration method remains essential for AR-based surgical navigation.

Despite the support provided by surgical navigation systems, surgeons are still required to manually define the entry and target points to establish the puncture trajectory. Zanello et al. [10] performed a retrospective analysis of complications caused by brain biopsy, revealing that the majority of iatrogenic cerebral hemorrhages arise from inadequate trajectory planning rather than intraoperative deviations from the surgical plan. Consequently, optimal path planning is the key to minimizing complications in brain biopsies. While manual path planning may be effective, it is a time-consuming and complex process requiring substantial expertise and experience [11]. Increasing research has focused on automated path planning methods. Shamir et al. [12,13,14] proposed a voxel-based risk map for keyhole neurosurgical path planning. This method defines intracranial arteries and ventricles as high-risk regions. The risk map is constructed by assigning a risk value to each voxel based on its Euclidean distance to these risk structures. The algorithm evaluates all candidate paths by accumulating the risk values of all voxels along the path and selects the trajectory with the lowest risk. However, this method only considers the distance to the closest risk structure when assessing the risk of each voxel, thereby potentially underestimating the risk of voxels that are simultaneously adjacent to multiple critical structures. Furthermore, the brute-force search approach increases computational burden. De León-Cuevas et al. [15] proposed a trajectory evaluation method using fuzzy logic. This approach integrates path length and voxel-wise risk through fuzzy rules. The fuzzy-optimized risk map is presented via a 3D visualization interface, supporting surgeons in selecting the safest path. However, its risk quantification capability is constrained by the fuzzy rules, and it requires the computation of risk for all potential paths. Villanueva-Naquid et al. [16] employed an enhanced risk map to generate candidate trajectories, combined with a genetic algorithm to search for the lowest-risk path. This method assesses the risk of each voxel as the aggregate of contributions from multiple adjacent high-risk structures. The use of a genetic algorithm effectively avoids exhaustive searches, decreasing the time by 99.9%. However, this method does not incorporate other clinical considerations (e.g., path length), which may limit its universality in practical surgical planning.

In summary, despite notable progress in AR-based surgical navigation, its routine clinical adoption remains limited by several fundamental constraints. Most existing systems depend heavily on precise marker placement or require time-consuming intraoperative setup procedures, which compromises robustness and disrupts surgical workflow efficiency. Moreover, the majority of current approaches lack automated path assessment and optimization, resulting in largely subjective selection of entry and target points. Consequently, critical trade-offs among path feasibility, surgical efficiency, and anatomical safety are not systematically evaluated. To address these limitations, we have developed an AR-based navigation system that combines multi-objective optimization (MOO) with hybrid registration. The system provides clinicians with real-time, accurate, and intuitive 3D navigation positioning information while performing automated path planning. The contributions of this work are summarized as follows:

(1) The proposed MOO method quantifies the clinical criteria for stereotactic brain biopsy procedures by establishing constraints and sub-objective functions that better reflect surgical guidelines.
(2) A hybrid registration approach is introduced to mitigate spatial drift inherent in conventional marker-based localization while maintaining a streamlined intraoperative workflow.

## 2. Materials and Methods

### 2.1. Overview of the Proposed System

**Figure 1:**
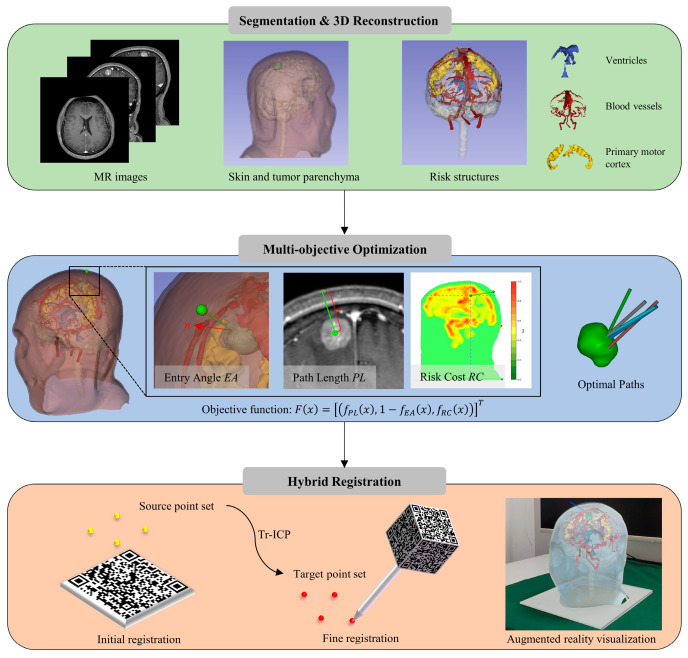
Workflow of the AR navigation system for stereotactic brain biopsy.

The overall workflow of our AR-based navigation system for stereotactic brain biopsy is illustrated in Figure 1. First, MRI data are acquired from the patient. Skin, tumor parenchyma, and high-risk structures are segmented using automated and semi-automated methods. Secondly, given the objective of path planning is to identify the path with the lowest risk, a multi-objective optimization problem is established based on clinical criteria. A constrained MOO model is formulated to solve for the optimal path. Finally, a hybrid registration method based on optical tracking is developed. Using the HoloLens 2 (Microsoft Corp., Redmond, WA, USA), the patient’s 3D anatomical structure model and the planned path are displayed in real time at the surgical site.

### 2.2. Preprocessing

Prior to path planning and 3D registration, segmentation of critical anatomical structures is required. The segmentation targets encompass three primary components: skin surface, which defines the skin entry point of the biopsy trajectory; tumor parenchyma, which determines the target point of the biopsy trajectory; and risk structures, which are crucial for path obstacle avoidance. Specific segmentation methods for each target are detailed as follows:

- Skin surface segmentation:

T1-weighted images (T1WI) are binarized using threshold segmentation to preliminarily separate the skull from the background. The Open–Close–Close–Shrink (OCCS) operation enhances skull boundary integrity by eliminating small holes and disconnected regions. Based on the acquired skull mask, external areas are contracted to define a thin layer near the surface as the candidate skin region.

- Tumor parenchyma segmentation:

Given the complex heterogeneity of tumors in multimodal MRI, the Wavelet-Guided Iterative Axial Factorization (WIAF) [17] model was employed to segment tumor parenchyma across T1WI, T2, and FLAIR.

- Risk structure segmentation:

Ventricles exhibit low signal intensity and regular morphology on T1WI, enabling segmentation via a thresholding method combined with region growing. Specialized clinicians used the 3D Slicer (5.8.1, 3D Slicer developer community, Cambridge, MA, USA) software platform to manually outline arterial and venous vascular structures on T1WI. The primary motor cortex (M1) was identified by threshold segmentation of fMRI t-maps to detect brain activation regions, which were then overlaid with spatial information registered to a standard brain atlas using Brainsuite (23a, University of Southern California and University of California Los Angeles, Los Angeles, CA, USA) software.

### 2.3. Multi-Objective Path Optimization

The primary objective of the brain biopsy path-planning problem is to find the path with the least risk. In clinical surgeries, the total length of the biopsy trajectory should be minimized to reduce the incidence of complications [15,16]. To ensure surgical feasibility, the angle between the biopsy needle and the skin incision plane should be as close to 90° as possible, while angles less than 20° may cause the needle to slip or bend [18]. Additionally, it is required that the biopsy path avoid the critical anatomical structures [19]. Based on the above clinical path planning principles, the brain biopsy path-planning problem is formulated as a multi-objective optimization model. Each path planning requirement is quantified as an objective subfunction or a constraint. Accordingly, the specific optimization objectives and constraints are defined as follows:

1. Path Length PL:

Path length is defined as the distance from the skin entry point to the target point on the tumor. A maximum path length of 90 mm is imposed as a constraint [15,16]. The calculation and constraints for path length are shown in Equation (1):

(1) $$\left\{\begin{array}{l} PL\left(x\right)=\sqrt{{\left(x_{T}-x_{E}\right)}^{2}+{\left(y_{T}-y_{E}\right)}^{2}+{\left(z_{T}-z_{E}\right)}^{2}}\\ PL\left(x\right)\leq 90mm \end{array}\right.,$$

where $\left(x_{E},y_{E},\;z_{E}\right)$ denotes the world coordinate of the skin entry point, and $\left(x_{T},y_{T},\;z_{T}\right)$ denotes the world coordinate of the target point.

2. Entry Angle EA:

The entry angle is defined as the supplementary angle between the normal vector of the skin entry point’s skin plane and the path direction vector. The minimum entry angle must not be less than 20°.The specific calculation and constraints for entry angle are shown in Equation (2):

(2) $$\left\{\begin{array}{l} EA\left(x\right)=\left|90-arccos\frac{\left(P_{E}-P_{T}\right)\cdot n_{E}}{\left\|P_{E}-P_{T}\right\|}\cdot \frac{180}{\pi }\;\right|\\ EA\left(x\right)\geq 20{}^{\circ} \end{array}\right.,$$

where $P_{E}$ and $P_{T}$ respectively denote the skin entry point and the target point, and $n_{E}$ is the normal vector of the skin cutting plane at the skin entry point.

3. Risk Cost RC:

To quantify the risk cost of trajectories, we construct a risk map across all brain voxels. A neurosurgeon assigns risk coefficients to each risk structure, representing the relative incidence of biopsy instruments crossing these areas [20]. Specifically, blood vessels, the M1, and the ventricles are assigned coefficients of 95%, 70%, and 45%, respectively. The risk value of any voxel is determined by its Euclidean distance from nearest-neighbor voxels within these risk structures, as defined in Equation (3):

(3) $$risk\left(v\right)=\sum_{S\in S}\sum_{i=1}^{N}r_{S}\cdot exp(-\frac{d^{2}}{2\sigma^{2}}),$$

where S is the set of all risk structures S, N is the number of nearest-neighbor voxels selected from each risk structure, $d_{S,i}$ denotes the physical distance between voxel v and its i nearest neighbor in risk structure S, $r_{S}$ denotes the risk coefficient assigned to structure S, and σ is the distance decay constant. In this work, N was set to 5 and σ to 10 mm.

The biopsy trajectory is a set of contiguous voxels that begins at an entry point and ends at a target point. Subsequently, the risk cost of the trajectory is defined as the sum of the risk values of all voxels constituting that path, as shown in Equation (4):

(4) $$RC\left(x\right)=\sum_{v_{i}\in U_{x}}risk\left(v_{i}\right),$$

where $U_{x}$ denotes the set of voxels comprising the path x, and $risk\left(v_{i}\right)$ denotes the risk value of the voxel $v_{i}$.

4. Restricted Areas RA:

We consider the following clinically restricted areas: the midline of the brain, which contains the median sagittal sinus vessels and is filled with cerebrospinal fluid [21]; blood vessels, to avoid intracranial hemorrhage [22]; ventricles, to preclude cerebrospinal fluid leakage that could lead to brain shift [23]; M1, to preserve postoperative motor function [24]; and the facial and ear areas, to minimize postoperative cosmetic impact [25]. The restricted puncture areas are defined as equation constraints for path optimization, as shown in Equation (5):

(5) $$U_{x}\cap U_{RA}=\phi ,$$

where $U_{x}$ denotes the set of voxels comprising the path x, and $U_{RA}$ denotes the set of voxels comprising the restricted area RA.

With the objective functions and constraints defined above, the MOO model for surgical path planning is established as shown in Equation (6):

(6) $$\begin{array}{c} MinimizeF\left(x\right)={\left[\left(f_{PL}\left(x\right),{1-f}_{EA}\left(x\right),f_{RC}\left(x\right)\right)\right]}^{T},\;\;x\in D\\ IC_{i}\left(x\right)\leq 0,i=1,2,\ldots ,n,\\ EC_{k}\left(x\right)=0,k=1,2,\ldots ,m, \end{array},$$

where the objective function $F\left(x\right)$ is defined to identify the puncture trajectory with the lowest risk. The decision variable x is a biopsy trajectory. The search space D is defined as the set of one-to-one mappings between candidate skin entry points and candidate target points. Inequality constraints $IC_{i}\left(x\right)$ are imposed on path length and entry angle, while equality constraints $EC_{k}\left(x\right)$ are used to restrict traversal through specific regions.

The optimal path is determined by minimizing the objective function $F\left(x\right)$ subject to the inequality and equality constraints. However, MOO problem faces the challenge of conflicting sub-objectives, making it difficult to achieve optimal outcomes across all sub-objectives simultaneously. To circumvent this inherent issue, we employ a vector-based method that utilizes the Non-dominated Sorting Genetic Algorithm II (NSGA-II) [26]. This algorithm treats the value of each sub-objective function as a feature of a single solution and optimizes them concurrently.

**Figure 2:**
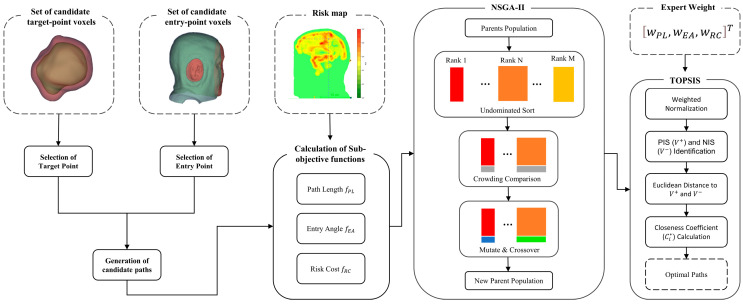
Flowchart of the MOO algorithm.

The flowchart of the MOO algorithm is illustrated in Figure 2. Given the MRI-based segmentations of critical anatomical structures and the risk map, the algorithm generates Pareto-optimal entry and target point pairs for biopsy path planning.

First, preprocessing is performed on the tumor and skin masks. According to expert consensus, a sufficient volume of lesion tissue is required in stereotactic biopsy to ensure reliable histopathological diagnosis. Specimens smaller than approximately 1 mm^3^ often result in diagnostic uncertainty [27]. Therefore, to ensure sufficient pathological tissue acquisition, we extracted voxels with a boundary thickness greater than 2 mm from the original tumor mask as candidate target points. To avoid generating paths through facial and auricular restricted areas, voxels outside the facial and auricular areas on the skin mask are extracted to form the candidate skin entry point set.

**Figure 3:**
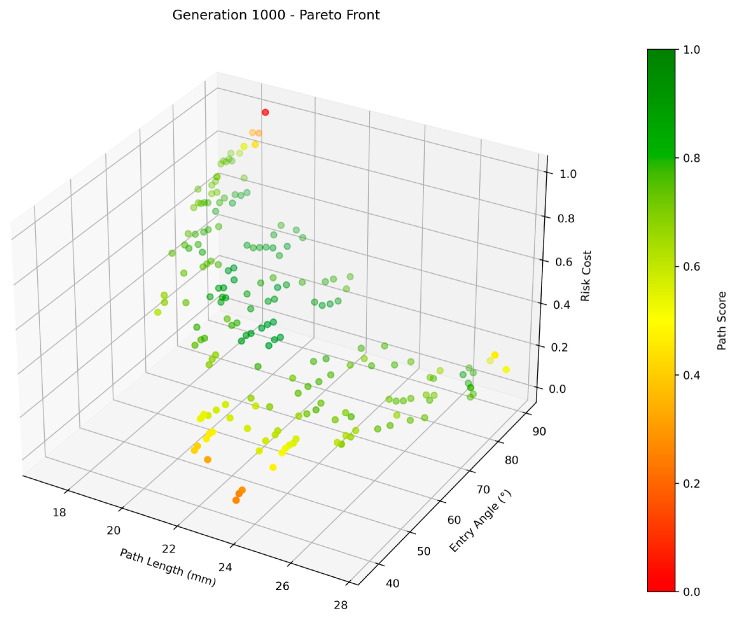
Pareto front with color-mapped quality assessments.

The initial population for NSGA-II is generated by randomly pairing candidate skin entry points with candidate target points. Any trajectory violating hard constraints is pre-filtered before being incorporated into the population. Each individual in the population represents a biopsy trajectory from a skin entry point to a target point, encoded as a 6-dimensional vector containing the coordinates of the entry and target voxels. Each individual corresponds to three sub-objective function values. Individuals are selected using non-dominated sorting and crowding distance to retain those with higher fitness. These high-fitness individuals, along with new individuals generated through crossover and mutation, form a new population to continue iterative evolution. In this work, the crossover process is performed by exchanging the skin entry points or target points between two parent individuals. Mutation updates the entry or target points of the parent individuals using a feasible neighbor identified by a K-nearest-neighbor search within the segmentation mask. After the final generation, the Pareto-optimal solution set is extracted and used for subsequent decision-making. The quality of these solutions is then evaluated based on an expert weight matrix and visualized through color mapping as shown in Figure 3.

Finally, the optimal paths are selected from the Pareto front using the Technique for Order Preference by Similarity to Ideal Solution (TOPSIS) method, incorporating expert weights. The expert weight vector $w={\left[w_{1},w_{2},\ldots ,w_{n}\right]}^{T}$ is derived from the expert priority matrix. The raw decision matrix is normalized and weighted to construct the weighted normalized matrix, as shown in Equation (7):

(7) $${\left[v_{ij}\right]}_{m\times n}={\left[w_{j}\frac{x_{ij}}{\sqrt{\sum_{i=1}^{m}x_{ij}^{2}}}\right]}_{m\times n},\forall i\in \left\{1,\ldots ,m\right\},j\in \left\{1,\ldots ,n\right\},$$

where $x_{ij}$ represents the j−th objective value of the i−th candidate path in the Pareto set, and m and n denote the number of candidate solutions and evaluation criteria, respectively.

Based on the decision matrix, the Euclidean distances of each candidate path to the positive and negative ideal solutions were subsequently computed. The comprehensive performance score $C_{i}^{*}$ for each candidate path is defined by a unified expression of relative closeness to the ideal solution, as shown in Equation (8):

(8) $$C_{i}^{*}=\frac{\sqrt{\sum_{j=1}^{n}{\left(v_{ij}-v_{j}^{-}\right)}^{2}}}{\sqrt{\sum_{j=1}^{n}{\left(v_{ij}-v_{j}^{+}\right)}^{2}}+\sqrt{\sum_{j=1}^{n}{\left(v_{ij}-v_{j}^{-}\right)}^{2}}},$$

where $v_{j}^{+}$ and $v_{j}^{-}$ denote the weighted normalized values of the positive-ideal and negative-ideal solutions, respectively.

The candidate path with the highest value $C_{i}^{*}$ was ultimately selected as the optimal trajectory for surgical planning.

### 2.4. Hybrid Registration

**Figure 4:**
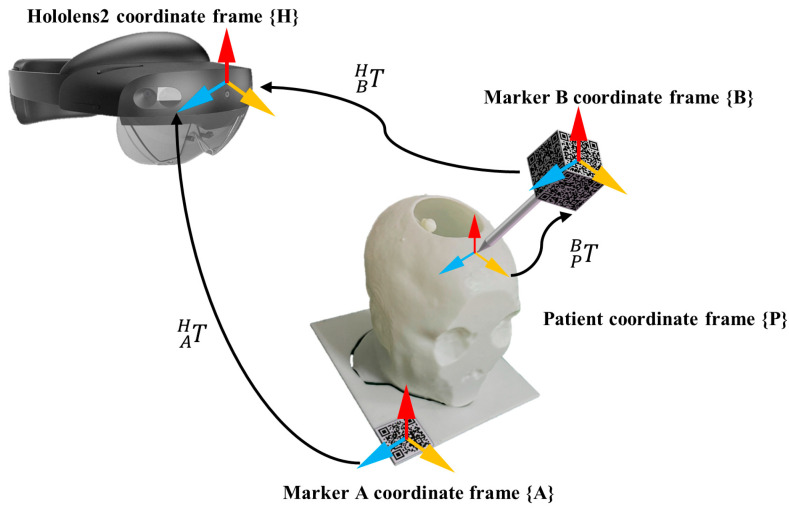
Coordinate systems and transformation matrices in the hybrid registration.

3D registration is the foundation, key, and difficulty of building an AR system [28]. To achieve accurate alignment between the virtual 3D anatomical model and the patient, we propose a hybrid registration framework that combines quick response (QR) codes with anatomical landmarks. The coordinate transformation for the hybrid registration is illustrated in Figure 4. The HoloLens 2 uses a spatial world anchor to define the Holenlens2 coordinate system {H}. After anatomy reconstruction and preoperative planning, all the medical models and path data are registered to {H}. We define the patient coordinate system {P} within the anatomical space of the patient’s head, while {A} and {B} denote the local coordinate systems associated with Marker A and Marker B, respectively.

Vuforia SDK is a development toolkit for detecting and tracking image targets with high accuracy, supporting the development of AR applications within the Unity platform [29]. The system uses two marker tools developed by Vuforia SDK. Marker A and Marker B are used for intraoperative localization and spatial drift compensation, respectively. Upon detection, the Vuforia Engine automatically identifies image features and estimates the poses of Marker A and Marker B relative to the {H}, denoted as TAH and TBH, respectively.

The hybrid registration process consists of initial registration and fine registration. The initial registration employs a QR code-based alignment method. Preoperatively, Marker A is manually fixed at a stable location on the patient or operating table, serving as an external reference marker within the surgical environment. The HoloLens 2 HMD identifies and tracks the real-time pose of the QR code, which determines the initial pose of the virtual 3D model in {H} via TAH. This step establishes a preliminary spatial correspondence between the virtual 3D model and the real surgical scene.

**Figure 5:**
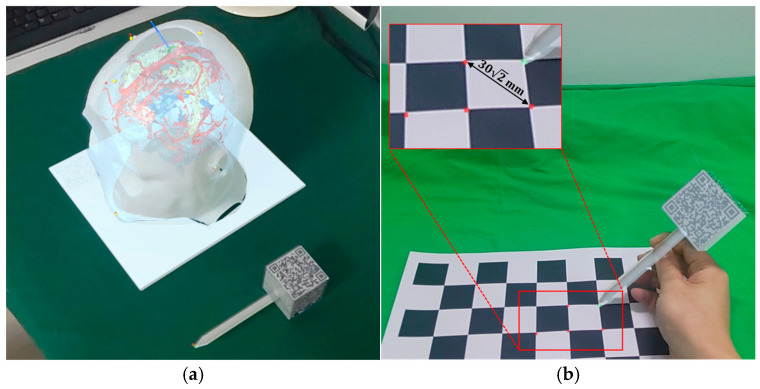
Spatial coordinate acquisition of reference points using Marker B: (**a**) Localization of anatomical landmarks on the phantom; (**b**) Localization of corner points from a high-precision checkerboard calibration board.

Prior to the procedure, anatomical landmarks (e.g., inner/outer canthus and nasal tip) were manually labelled on the reconstructed 3D mesh. In regions lacking distinct biological features, supplementary reference markers were strategically defined as virtual fiducials. To compensate for the inaccuracies of the initial registration, Marker B facilitates fine registration by localizing these predefined landmarks on the patient through multiple image target tracking. Marker B is mounted on a handheld probe and defines a local coordinate system {B} centered at its geometric origin. The transformation matrix TPB is derived from the real-time pose of the probe’s needle tip relative to the coordinate system {B}. When the operator aligns the probe’s needle tip with a landmark and maintains the position beyond a predefined duration, the system automatically records the needle tip’s coordinates in the coordinate system {B}. A red sphere is then generated at the sampled position to provide visual feedback, as shown in Figure 5.

Subsequently, these coordinates centered on Marker B are transformed into the coordinate system {H} via TBH. The coordinates of the acquired patient anatomical landmarks in the coordinate system {H} are given by Equation (9):

(9) PH=TBHTPPPB,

where $P_{H}$ denotes the coordinates of the anatomical landmarks in the coordinate system {H}, and $P_{P}\;$denotes the coordinates of the anatomical landmarks in the patient coordinate system {P}.

The 3D coordinates of feature points acquired from the patient are defined as the target point set ${Set}_{P}=\{P_{H,i}\}$. Correspondingly, the feature point set on the virtual model constitutes the source point set ${Set}_{A}=\{A_{H,i}\}$. The Trimmed Iterative Closest Point (Tr-ICP) algorithm is utilized to minimize the sum of squared distances between the target point set and the source point set. The obtained optimal transformation is shown in Equation (10):

(10) TAP=min∑i=1N∥RAH,i+t−PH,i∥2,

where R is the rotation matrix, and t is the translation vector.

Finally, the optimal transformation is applied to the initial pose of the virtual model. Through a secondary pose transformation, precise alignment between the virtual model and the patient’s anatomical structures is achieved. Since Marker A remains fixed relative to the patient, the system can continuously track intraoperative patient or operating table movements. The system dynamically updates the pose of the registered virtual model, which effectively compensates for navigation inaccuracies caused by patient motion during surgery.

After the registration process, the operator can visualize the 3D anatomical structures and the planned path overlaid on the patient through the HoloLens 2, facilitating an intuitive surgical navigation.

### 2.5. Experiment

#### 2.5.1. Expert Study

**Figure 6:**
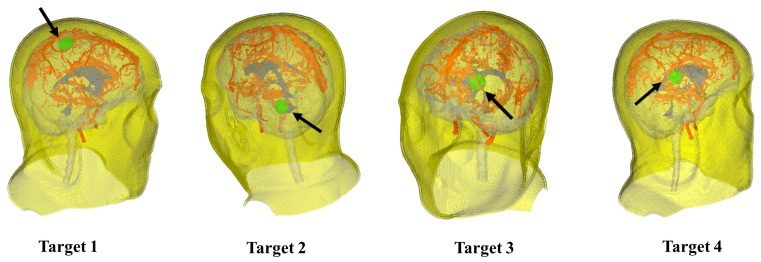
Four common locations (in green) of brain tumors used during evaluation.

We utilized the IEEE Visualization Contest 2010 Case 2 dataset [30], which comprises multi-modal MRI data, including T1WI, T2, FLAIR, and fMRI. The T1WI volume serves as the spatial reference for this study, featuring a matrix size of 512 × 512 with 176 slices and a voxel spacing of 0.49 × 0.49 × 1.0 mm^3^. All other multimodal data were linearly co-registered to this T1WI coordinate space. Based on neurosurgical consensus, four typical brain tumor locations were established. Target 1 represents the actual tumor location in the patient, while the remaining tumors were artificially placed within the scene, as shown in Figure 6. Target 1 is situated in the precentral gyrus, in close proximity to major blood vessels. Target 2 is located in the left temporal lobe. Target 3 lies adjacent to the cerebral ventricle within the right parietal lobe, and Target 4 is positioned within the left prefrontal cortex.

For each target, the proposed MOO method was executed 20 times to automatically generate the optimal path. The MOO algorithm was implemented in Python 3.11. The NSGA-II was configured for use with a population of 200 individuals in a total of 1000 generations. The probability of crossover and mutation was configured to 100% and 30%, respectively. The algorithm terminated after reaching the maximum generation limit. To identify the optimal paths, TOPSIS was employed with a fixed weight vector of [0.35, 0.2, 0.45]. All computational tasks were conducted on a workstation equipped with an Intel Core i7-12700H CPU (2.3 GHz) and 16 GB RAM.

**Figure 7:**
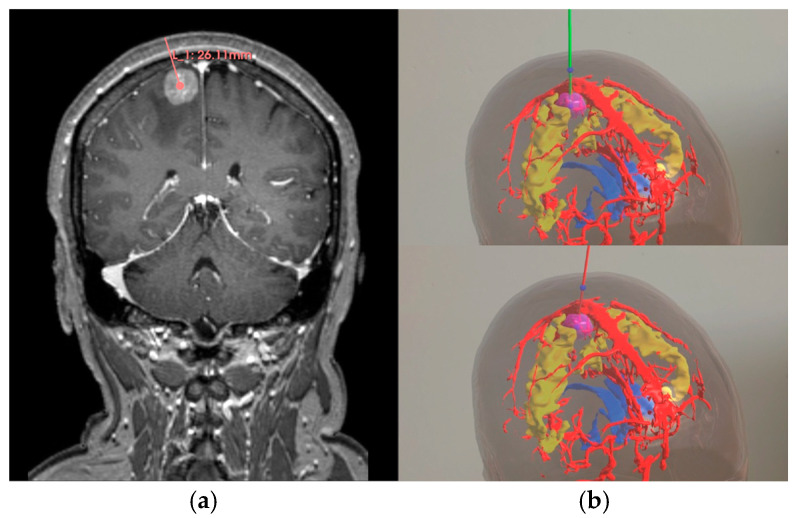
Two manual path planning methods: (**a**) Manual path planning based on 2D images (in pink); (**b**) Interactive path planning using AR (safe mode in green and risk mode in red).

To evaluate the quality of paths generated by the MOO model, an experienced neurosurgeon and three novice surgeons were recruited to perform manual path planning and AR-based interactive path planning, as shown in Figure 7. For each target, the experienced neurosurgeon repeated the planning three times, whereas each novice surgeon performed one planning trial. Manual path planning requires the surgeon to manually select skin entry points and target points according to the standard of care. We developed an AR-based interactive path planning system [31]. Users wearing the HoloLens 2 HMD could clearly visualize the patient’s 3D anatomical model. When the operator manually selects a target location, the system automatically generates a path based on the skin entry point closest to the target. Subsequently, users can interactively adjust the path via gesture input, enabling rotation operations centered on the target. During this process, path color feedback is triggered if the trajectory intersects restricted areas or violates the length or angle constraints.

During the experiment, four indicators were used to evaluate paths. Specifically, path length, entry angle, and risk cost are defined in Section 2.3. The minimum Euclidean distance from the path to the risk structure serves as the safe margin metric, as shown in Equation (11):

(11) $$d_{min}=\underset{(x,y,z)\in P,(u,v,w)\in S}{min}\sqrt{(\Delta_{x}(x-u){)}^{2}+(\Delta_{y}(y-v){)}^{2}+(\Delta_{z}(z-w){)}^{2}}\;,$$

where $\left(x,y,z\right)$ denotes the voxel coordinates of an arbitrary voxel within along the path P. $\left(u,v,w\right)$ denotes the voxel coordinates of an arbitrary voxel within the risk structure S. $\Delta_{x}$, $\Delta_{y}$ and $\Delta_{z}$ denote the voxel spacing along the three spatial directions of the MRI volume.

#### 2.5.2. Phantom Experiment

**Figure 8:**
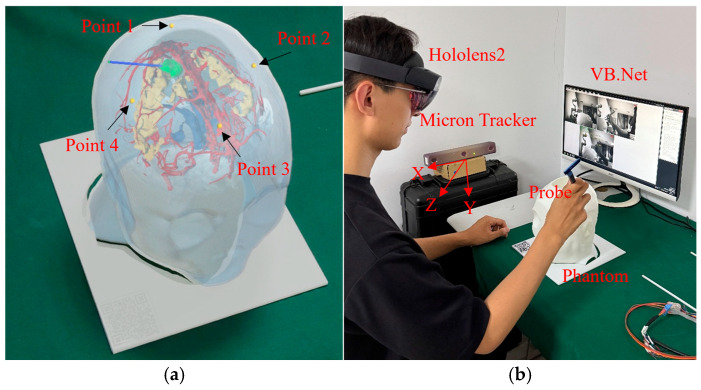
Materials of phantom experiment: (**a**) Reference points (yellow spheres) for registration accuracy testing; (**b**) Subjects recording spatial coordinate information using Micron Tracker.

To measure the registration errors of the AR navigation system, we conducted 20 phantom experiments. The phantom used in the experiments was obtained through 3D reconstruction and 3D printing, preserving the precise relative positions of anatomical structures. Four yellow spheres were placed on the head model to serve as reference points for accuracy testing, as shown in Figure 8a. Micron Tracker (ClaroNav, North York, ON, Canada) served as the ground truth spatial coordinate acquisition device, achieving an accuracy of 0.2 mm after calibration. The spatial coordinates of the point were obtained by maintaining the optical marker within the trinocular camera’s detection range and aligning the probe tip with the target point, as shown in Figure 8b.

First, the operator wore the HoloLens 2 HMD and initiated the AR navigation system. Initial registration was performed by gazing at Marker A. The appearance of a virtual 3D model near Marker A confirms successful initial alignment. The Micron Tracker was used to obtain the spatial coordinates of reference points on both the phantom and the virtual model. Subsequently, fine registration was performed using Marker B. The Micron Tracker was used again to acquire updated coordinates of the reference points. The Fiducial Registration Error (FRE) of both registrations was calculated to evaluate the accuracy of the hybrid registration. FRE is defined as the Euclidean distance between the reference points of the virtual model and the phantom, as shown in Equation (12):

(12) $$FRE=\sqrt{{\left(x_{1}-x_{2}\right)}^{2}+{\left(y_{1}-y_{2}\right)}^{2}+{\left(z_{1}-z_{2}\right)}^{2}},$$

where $\left(x_{1},y_{1},z_{1}\right)$ denotes the coordinates of the reference point on the virtual model, and $\left(x_{2},y_{2},z_{2}\right)$ denotes the coordinates of the corresponding reference point on the phantom.

After registration, the Target Registration Error (TRE) was additionally measured at the planned biopsy target. TRE is defined as the Euclidean distance between the target point of the virtual model and the phantom, as given in Equation (13):

(13) $$TRE=\sqrt{{\left(x_{1}-x_{2}\right)}^{2}+{\left(y_{1}-y_{2}\right)}^{2}+{\left(z_{1}-z_{2}\right)}^{2}},$$

where $\left(x_{1},y_{1},z_{1}\right)$ denotes the coordinates of the target point on the virtual model, and $\left(x_{2},y_{2},z_{2}\right)$ denotes the coordinates of the corresponding target point on the phantom.

## 3. Results

### 3.1. Path Evaluation

**Figure 9:**
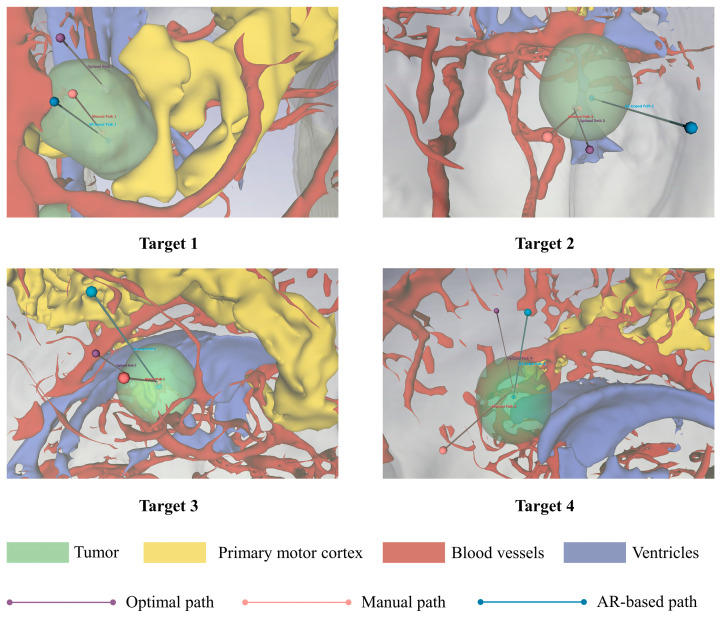
3D visualization of the path planning results.

A comparative analysis was conducted across manual path planning, AR-based path planning and the MOO algorithm. Representative path planning results were visualized in three dimensions, as shown in Figure 9. The top-ranked path obtained by the MOO algorithm is considered the optimal path, marked with purple lines in each image. Manually planned paths and those derived from AR-based path planning are labeled with pink and blue lines, respectively. The path planning process considers the feasibility of a cylindrical puncture zone centered at the tumor target point with a 2 mm radius. Path planning results are presented as line segments within the 3D head reconstruction, aiming to clearly visualize the entry and target points, along with the spatial extent of key anatomical structures for the surgeons. Each image in Figure 9 is derived from the 3D reconstruction results. Within the 3D reconstruction, certain vital organs are rendered using pseudo-color processing techniques. The anatomical structures are visualized with varying transparency and color intensity, including the tumor (light green), primary motor cortex (yellow), blood vessels (deep red), and ventricles (deep blue).

**Figure 10:**
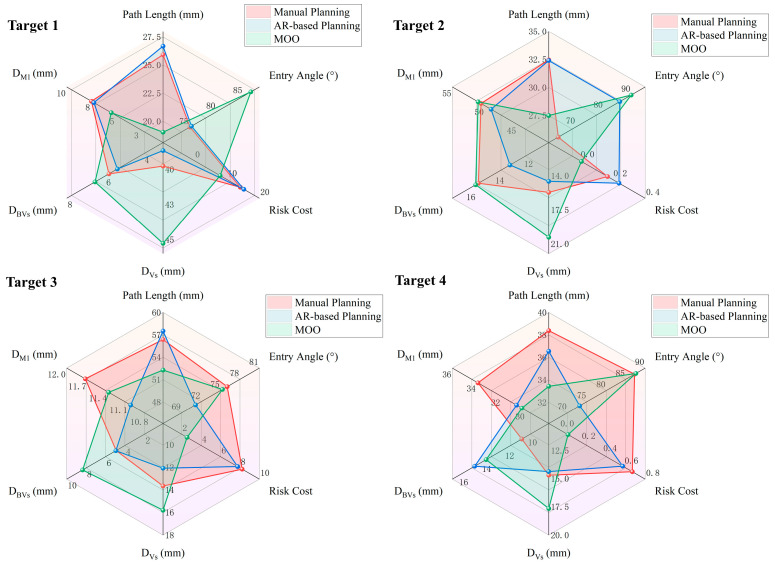
Radar chart of the path evaluation.

The performance of the three planning methods was evaluated across six metrics defined in Section 2.5.1. Table 1 shows the quantitative outcomes of the path evaluation derived from the multi-objective optimization functions, while Table 2 summarizes the safety distance results. The comparative results across all six metrics are illustrated in Figure 10. In the radar chart, diminished values signify superior performance for Path Length and Risk Cost, while elevated values suggest enhanced performance for the other metrics.

Subsequently, a linear mixed-effects model was applied, with Method as a fixed effect and Target as a random effect. Table 3 shows the estimated marginal means and standard error, with *p*-values adjusted using Bonferroni correction for multiple comparisons.

The results indicated that the MOO algorithm yielded significant improvements across nearly all metrics. Specifically, it reduced the mean path length by 13.6% compared to manual planning and by 13.7% compared to AR-based planning. Similarly, the MOO algorithm dramatically reduced the mean risk cost by 54.3% and 55.4% compared to manual and AR-based methods, respectively. Simultaneously, the mean entry angle of the paths generated by this method was significantly improved by 12.4% and 11.1% compared to manual planning and AR-based planning, respectively. Furthermore, the MOO method significantly increased the minimum distance to the ventricles by 18.2% and 24.3% compared to manual and AR-based methods, respectively. The minimum distance to blood vessels also significantly increased by 20.6% and 15.8%. Regarding the minimum distance to M1, the MOO method performed comparably to the AR-based planning without a significant difference, though it exhibited a slight 5.3% decrease compared to the manual planning. Compared with manual planning, the AR-based method significantly reduced the minimum distances to blood vessels and M1 by 4.96% and 5.44%, respectively, while no significant differences were observed in the remaining metrics.

Beyond the quantitative evaluation of path quality, we further assessed planning efficiency. The mean time for manual planning was 7.91 ± 1.04 min, compared with 6.65 ± 1.59 min for AR-based planning. Notably, the MOO method reduced the mean time to 2.98 ± 0.08 min.

### 3.2. Phantom Experiment Results

**Figure 11:**
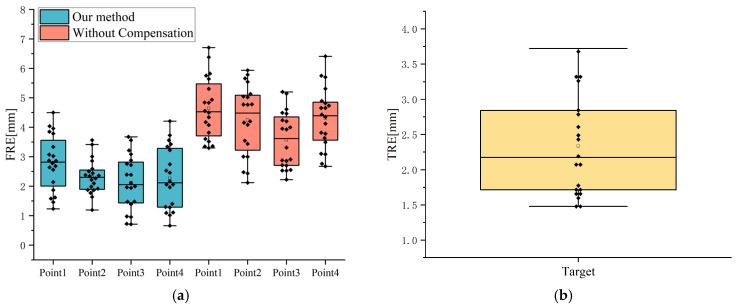
Phantom experiment results: (**a**) FRE; (**b**) TRE. In the box plots, the box boundaries represent the 25th and 75th percentiles, while the horizontal lines, squares, and diamonds represent the median, mean, and individual data points, respectively.

Phantom experiment results are shown in Figure 11. Using only QR code-based registration for initial alignment yielded a mean FRE of 4.19 ± 1.11 mm, with a maximum value of 6.70 mm and a minimum value of 2.12 mm. The FRE for Point1 was 4.64 ± 1.03 mm, for Point2 it was 4.24 ± 1.19 mm, for Point3 it was 3.57 ± 0.95 mm, and for Point4 it was 4.31 ± 1.03 mm.

After spatial drift compensation using anatomical landmarks, the mean FRE was 2.37 ± 0.91 mm, with a maximum of 4.50 mm and a minimum of 0.66 mm. The FRE for Point1 was 2.79 ± 0.94 mm, for Point2 it was 2.31 ± 0.58 mm, for Point3 it was 2.12 ± 0.92 mm, and for Point4 it was 2.27 ± 1.05 mm.

The proposed hybrid registration significantly reduced registration error compared to the uncompensated condition. The mean FRE decreased from 4.19 ± 1.11 mm to 2.37 ± 0.91 mm (mean difference: 1.82 ± 0.91 mm). A linear mixed-effects model demonstrated a significant reduction in FRE (β = −1.818, z = −16.23, *p* < 0.001), with Trial specified as a random effect.

As shown in Figure 11b, the mean TRE was 2.34 ± 0.71 mm, with a maximum of 3.72 mm and a minimum of 1.48 mm. Meanwhile, the mean hybrid registration time was 2.63 ± 0.36 min.

## 4. Discussion

Stereotactic brain biopsy is a critical procedure in neurosurgery for the pathological diagnosis of intracranial lesions. The accuracy of trajectory planning and spatial registration directly determines surgical safety and diagnostic yield of biopsy. However, in clinical practice, the subjectivity of preoperative biopsy path planning and registration drift remain core challenges limiting the widespread clinical adoption of AR navigation. In this study, we proposed an AR navigation system for brain biopsy that integrates automated path planning with hybrid registration. This integrated solution is designed to streamline the surgical workflow and mitigate surgical risks. Preoperatively, the system automatically generates the optimal paths to assist surgeons in surgical planning. With the help of this system, surgeons can visualize both the 3D anatomical model and the preplanned trajectory directly in the working surgical view. Intraoperatively, the system allows surgeons to operate the neuronavigation system at a more natural posture and without shifting views.

The proposed MOO model establishes objective functions and constraints based on clinical criteria, comprehensively considering path length, entry angle, and risk cost. This approach simulates clinical surgical decision-making, translating empirical planning into a data-driven automated optimization solution. In the expert study, we compared the performance of three path planning methods across six metrics. The finding that novices achieved comparable performance to an expert in four metrics within a shorter time suggests that the AR interface effectively bridges the spatial cognition gap. Traditional 2D-based planning requires surgeons to mentally reconstruct complex 3D anatomy from sequential slices, which requires specialized knowledge and clinical experience. By providing an intuitive, stereoscopic visualization of the brain structures, AR technology offloads this cognitive burden, allowing novice surgeons to identify safe trajectories as effectively as experienced surgeons. However, the observed decreases in minimum distance to blood vessels and M1 indicate that visual guidance alone is insufficient to replicate the nuanced risk-avoidance intuition of a senior surgeon.

Path evaluation results demonstrate that the MOO algorithm significantly outperformed both manual and AR-based path planning across five evaluation indicators (*p* < 0.01), except for minimum distance to M1. Although both risk cost and safety distance quantify trajectory risk, the MOO algorithm demonstrated distinct performance patterns across these metrics. This discrepancy stems from the different evaluation mechanisms between the objective function and the distance metric. The MOO algorithm evaluates the risk cost of the path by accumulating discrete voxel risk values along the path. In contrast, minimum distance to M1 represents a continuous spatial measurement from the closest single point on the path to the boundary of the risk structure. Consequently, while a specific short segment of the MOO path might pass closer to M1 and thus yield a lower minimum distance, the significantly reduced risk cost demonstrates that most voxels along the generated path are constrained within low-risk regions. In addition, the results indicate that the MOO algorithm does not demonstrate clear superiority over manual planning for certain tumor locations. For Target 1 and Target 4, the MOO paths reduced the minimum distance to M1 by 2.04 mm and 3.6 mm compared to manual planning, respectively. Meanwhile, path length was shortened by 6.62 mm and 4.95 mm, and the minimum distance to vessels increased by 0.72 mm and 3.11 mm, respectively. The reduction in risk cost can be attributed to the shorter path length and the increased distance from vessels with high-risk coefficients. Most importantly, the minimum distances to M1 maintained by the MOO paths remain substantially larger than the universally accepted clinical safety margin of 2 mm. For Target 3, the MOO paths decreased the mean entry angle to 0.74° compared to the manually planned paths. However, it demonstrated clear advantages in terms of path length and risk cost. Notably, the minimum distance to blood vessels increased from 4.92 mm to 8.37 mm, substantially improving risk avoidance. Clinically, a modest compromise in entry angle is generally acceptable when it results in a trajectory with lower risk. Beyond path quality, the MOO algorithm requires a mean time of 2.98 ± 0.08 min. Compared with the manual and AR-based methods, it was more efficient and automated, potentially contributing to improved surgical workflow.

It should be noted that the optimization outcomes remain sensitive to the weights defined by the surgeons. While this allows for clinical flexibility, such reliance on human input may lead to variability in path quality depending on the practitioner’s specific priorities. Furthermore, our path planning algorithm has yet to integrate intraoperative real-time imaging (e.g., ultrasound or MRI) for brain shift compensation. Another limitation of the method is the lack of constraints to avoid cerebral sulci or to ensure the path within the white matter. Future research will integrate intraoperative imaging for dynamic model updating and extend the optimization formulation with additional constraints and objectives.

To address registration errors caused by patient movement and spatial drift during surgery, a hybrid registration method combining QR codes and anatomical landmarks is proposed. In the phantom experiment, the mean FRE was 2.37 ± 0.91 mm, representing a 43.2% improvement in accuracy compared to traditional augmented reality navigation relying on a single marker. This improvement demonstrates that the hybrid registration method effectively mitigates spatial drift induced by QR code tracking. The mean TRE of the system was 2.34 ± 0.71 mm. Nevertheless, Zhou et al. [9] achieved a mean TRE of 1.41 mm using markerless spatial registration, with a mean registration time of 6.02 min, indicating that further improvements are still necessary. The suboptimal registration accuracy may stem from the development of markers using the Vuforia SDK, which is susceptible to variations in recognition distances, image feature detection angles, and ambient lighting [32]. These conditions can cause model jitter and instability. In addition, anatomical landmark recognition remains manually performed. The operator’s field of view is restricted by wearing the HMD, which consequently introduces errors into manual calibration methods. Future work will explore deep learning-based automated anatomical landmark recognition to streamline the registration process and enhance spatial registration accuracy.

In terms of procedural efficiency, the total time required for the registration process was optimized to 2.63 ± 0.36 min. Traditional stereotactic navigation, especially frame-based stereotaxy, often requires more extensive setup and manual registration steps, which can be time-consuming. By providing a streamlined, non-invasive registration process within the AR environment, the hybrid registration reduces the technical complexity for the surgical team.

From a clinical perspective, the accuracy of the system is sufficient for guiding biopsies of large-volume lesions particularly when located in non-eloquent brain regions [8]. In these scenarios, the lesion’s spatial extent provides an adequate safe buffer for the observed registration error. However, for precision-sensitive cases involving small targets or lesions in critical structures like the thalamus or brainstem, the registration accuracy remains insufficient for such delicate interventions. In these scenarios, even minor deviations exceeding clinical tolerance can lead to non-diagnostic tissue sampling or severe neurological deficits. The proposed system should serve as a supplementary tool for situational awareness.

Nevertheless, the validation of this work was conducted on a limited dataset and participants, along with phantom-based experiments under controlled conditions. Although the results demonstrate the technical feasibility and potential advantages of the proposed framework, they should be interpreted as preliminary findings.

## 5. Conclusions

In summary, we propose an AR navigation system for stereotactic brain biopsy that integrates preoperative automated path planning with intraoperative 3D registration. The system constructs a MOO model to quantify clinical criteria, selecting optimal paths by solving for Pareto-optimal solutions. Concurrently, it employs a hybrid registration method to achieve high-precision intraoperative path navigation and 3D visualization of critical anatomical structures. Experimental results indicate that paths planned by MOO entail lower risk compared to the paths manually planned by clinical surgeons. Furthermore, the hybrid registration approach demonstrates higher accuracy than traditional QR code-based registration within the AR framework, while maintaining a relatively streamlined registration workflow.

## Figures and Tables

**Table 1:** Results of the path evaluation on multi-objective optimization functions.

Target	Method	*PL* (mm)	*EA* (°)	*RC*
Target 1	Manual	25.92 ± 0.22	75.11 ± 1.87	14.16 ± 1.43
	AR-based	26.68 ± 1.65	75.39 ± 3.89	15.23 ± 1.55
	MOO	**19.03 ± 0.24**	**86.42 ± 2.51**	**7.96 ± 0.42**
Target 2	Manual	32.46 ± 0.50	63.55 ± 3.62	0.17 ± 0.04
	AR-based	32.40 ± 1.66	85.82 ± 2.37	0.24 ± 0.10
	MOO	**27.51 ± 0.42**	**89.95 ± 0.18**	**0.01 ± 0.00**
Target 3	Manual	56.37 ± 0.87	**76.03 ± 0.54**	8.23 ± 0.52
	AR-based	57.51 ± 0.24	71.13 ± 1.33	7.76 ± 0.29
	MOO	**52.28 ± 0.23**	75.29 ± 0.67	**2.57 ± 0.17**
Target 4	Manual	38.38 ± 0.48	87.27 ± 2.51	0.68 ± 0.37
	AR-based	36.55 ± 1.45	73.09 ± 1.50	0.59 ± 0.34
	MOO	**33.43 ± 0.97**	**87.60 ± 1.66**	**0.08 ± 0.05**

Bold values indicate the best performance among the compared methods.

**Table 2:** Results of the path evaluation on safety distances.

Target	Method	D_Vs_ (mm)	D_BVs_ (mm)	D_M1_ (mm)
Target 1	Manual	39.77 ± 1.08	5.81 ± 0.86	**7.46 ± 1.27**
	AR-based	38.67 ± 1.46	5.37 ± 0.16	7.21 ± 0.67
	MOO	**45.27 ± 0.06**	**6.53 ± 0.18**	5.42 ± 0.09
Target 2	Manual	15.70 ± 0.41	15.09 ± 0.83	50.58 ± 2.63
	AR-based	14.57 ± 1.77	12.90 ± 0.52	49.02 ± 0.71
	MOO	**20.32 ± 0.64**	**15.28 ± 1.02**	**51.02 ± 0.93**
Target 3	Manual	13.66 ± 0.62	4.92 ± 0.09	**11.73 ± 2.74**
	AR-based	12.08 ± 2.29	4.95 ± 0.71	11.07 ± 0.65
	MOO	**15.81 ± 0.55**	**8.37 ± 0.32**	11.39 ± 0.13
Target 4	Manual	14.70 ± 1.27	10.45 ± 0.89	**33.90 ± 0.79**
	AR-based	14.38 ± 0.39	14.55 ± 0.35	30.74 ± 1.08
	MOO	**17.66 ± 0.97**	**13.56 ± 0.88**	30.30 ± 0.67

D_Vs_, D_BVs_, and D_M1_ denote the minimum 3D Euclidean distance to the ventricles, vessels, and M1, respectively. Bold values indicate the best performance among the compared methods.

**Table 3:** Linear mixed-effects model analysis of three planning methods.

Metrics	Manual Planning	AR-Based Planning	MOO
PL (mm)	38.28 ± 6.96	38.28 ± 6.96	33.06 ± 6.95 **††
EA (°)	75.49 ± 2.92	76.36 ± 2.92	84.82 ± 2.69 **††
RC	5.81 ± 2.24	5.96 ± 2.24	2.65 ± 2.22 **††
D_Vs_ (mm)	20.96 ± 6.76	19.92 ± 6.76 *	24.76 ± 6.75 **††
D_BVs_ (mm)	9.07 ± 2.14	9.44 ± 2.14	10.94 ± 2.12 **††
D_M1_ (mm)	25.92 ± 10.20	24.51 ± 10.20 **	24.53 ± 10.19 **

* Significant difference compared to manual planning (*p* < 0.05). ** Significant difference compared to manual planning (*p* < 0.01). †† Significant difference between MOO and AR-based planning (*p* < 0.01).

## Data Availability

The original data presented in the study are openly available in the IEEE Visualization Contest 2010 at https://sciviscontest.ieeevis.org/2010/data.html, accessed on 24 November 2025. The source code of this work is publicly available at https://github.com/characterz0829-prog/AR-based-Navigation-for-Stereotactic-Brain-Biopsy, accessed on 9 January 2026.
